# Vacuum Ultraviolet Field Emission Lamp Consisting of Neodymium Ion Doped Lutetium Fluoride Thin Film as Phosphor

**DOI:** 10.1155/2014/309091

**Published:** 2014-09-11

**Authors:** Masahiro Yanagihara, Takayuki Tsuji, Mohd Zamri Yusop, Masaki Tanemura, Shingo Ono, Tomohito Nagami, Kentaro Fukuda, Toshihisa Suyama, Yuui Yokota, Takayuki Yanagida, Akira Yoshikawa

**Affiliations:** ^1^Nagoya Institute of Technology, Gokiso-cho, Showa-ku, Nagoya, Aichi 466-8555, Japan; ^2^Department of Materials, Manufacturing & Industrial , Faculty of Mechanical Engineering, Universiti Teknologi Malaysia (UTM), 81310 Skudai, Johor, Malaysia; ^3^Tokuyama Corporation, Kasumigaseki Common Gate West Tower 2-1, Kasumigaseki 3-chome, Chiyoda-ku, Tokyo 100-8983, Japan; ^4^Institute for Materials Research, Tohoku University, 2-1-1 Katahira, Aoba-ku, Sendai 980-8577, Japan; ^5^Kyushu Institute of Technology, 2-4 Hibikino, Wakamatsu-ku, Kitakyushu 808-0196, Japan

## Abstract

A vacuum ultraviolet (VUV) field emission lamp was developed by using a neodymium ion doped lutetium fluoride (Nd^3+^ : LuF_3_) thin film as solid-state phosphor and carbon nanofiber field electron emitters. The thin film was synthesized by pulsed laser deposition and incorporated into the lamp. The cathodoluminescence spectra of the lamp showed multiple emission peaks at 180, 225, and 255 nm. These emission spectra were in good agreement with the spectra reported for the Nd^3+^ : LuF_3_ crystal. Moreover, application of an acceleration voltage effectively increased the emission intensity. These results contribute to the performance enhancement of the lamp operating in the VUV region.

## 1. Introduction

Vacuum ultraviolet (VUV) light has been used in numerous fields, such as cleaning, surface modification, and sterilization, because short wavelength light with high photon energy is capable of breaking strong chemical bonds [1–3]. Therefore, performance improvements of VUV lamps contribute to the progress of these applications. The VUV gas lamp has widely been used [4–6] but presents limited stability, lifetime, and size. VUV lamps using a solid-state phosphor have attracted considerable attention as alternate light sources because they exhibit less deterioration, less fluctuation, and higher density than gas lamps [7, 8]. These lamps require wide band gap materials but few solid-state phosphors have substantial band gaps. Group III nitrides are suitable because they present a direct transition type band structure with a wide band gap [9, 10]. However, even when using AlN, which emits light at a relatively short wavelength, the operating wavelength was limited to deep UV region [9, 11–13]. The wide band gap of diamond can be applied to UV but not to VUV lamps [14]. On the other hand, some fluorides have band gaps that are sufficiently wide to enable light emission in the VUV region [15, 16]. Fluoride composite materials have been widely studied as laser materials, scintillation materials, and optical materials because of their extremely wide band gap [17–24]. Specifically, a KMgF_3_ thin film acting as a solid-state phosphor and carbon nanofiber (CNF) field electron emitter has previously been incorporated into a VUV lamp [25]. The emission spectra from the lamp showed two emission peaks at 155 and 180 nm in the 140–200 nm wavelength range, showing that solid-state phosphors can be exploited in VUV lamps.

Neodymium ion doped lutetium fluoride (Nd^3+^ : LuF_3_), whose cathodoluminescence (CL) efficiency is almost equivalent to KMgF_3_, was selected as a phosphor to develop a new VUV lamp. This lamp also consisted of CNFs field electron emitters. Among Nd^3+^ ion doped fluoride materials that emit VUV light, such as Nd^3+^ : LuF_3_, Nd^3+^ : LaF_3_, and Nd^3+^ : LuLiF_4_ [26–28], Nd^3+^ : LuF_3_ single crystals have reported the highest X-ray excited luminescence conversion efficiency [26]. However, large Nd^3+^ : LuF_3_ single crystals have proven difficult to grow because of the occurrence of a hexagonal to orthorhombic phase transition (ca. 950°C) during the crystal growth process [26]. The stress caused by this structural reconfiguration results in crack formation in Nd^3+^ : LuF_3_ single crystals. In contrast, growth of thin film suppresses these cracks owing to reducing stress by depositing small particles. For this reason, we fabricated Nd^3+^ : LuF_3_ thin film by pulsed laser deposition (PLD) to deposit small particles. In addition, PLD has produced fewer chemical composition discrepancies between source targets and deposited thin films. Consequently, the fabrication of fluoride thin films by PLD does not require the utilization of the toxic fluorine gas [29].

## 2. Experimental Methods

### 2.1. Thin Film Fabrication

The target was prepared by pressing a 1 : 9 NdF_3_-LuF_3_ powder mixture. A (001)-oriented MgF_2_ crystal (20 mm × 20 mm × 0.5 mm) mounted on a rotating holder was used as a substrate and was maintained at 400°C during PLD. This substrate temperature was chosen because previous experiments on the growth of Nd^3+^ : LaF_3_ thin films showed that substrate heating improved crystalline quality and VUV luminescence quantum efficiency and resulted in optimal performance at 400°C [27]. The thin film was deposited by irradiating the Nd^3+^ : LuF_3_ target with the third harmonics of a Nd : YAG laser (355 nm in wavelength). The 2 mm diameter laser spot was focused on the target at a fluence of 2.5 J/cm^2^ and a repetition rate of 10 Hz. The deposition was carried out for 8 h at an average pressure of 3 × 10^−4^ Pa without atmosphere control.

### 2.2. Field Emission Lamp Construction

**Figure 1:**
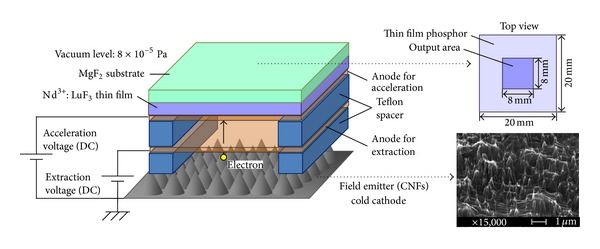
Schematic diagram of VUV field emission lamp. SEM image of CNFs is shown in the insert.

CNFs were grown by bombarding a grassy carbon substrate with Ar^+^ at room temperature [30–32]. The ion beam, which had a diameter of 6 cm, was set at an incident angle of 45° and energy of 1 keV, respectively. The length and diameter of CNFs were 0.3–2 and 20 mm, respectively, with an approximate density of 5 × 10^8^ cm^−2^.  Figure 1 shows the schematic of the lamp. In addition to the CNFs and the thin film, the lamp contained two copper mesh electrodes with a mesh width of 0.1 mm. Two teflon spacer plates were used to prevent short circuits and provide space for electron acceleration. A 200 *µ*m thick spacer was placed between CNFs and a copper electrode and a 5 mm thick spacer was placed between the two copper electrodes. In this lamp, electrons were emitted from CNFs using the extraction voltage and accelerated toward the thin film using the acceleration voltage. VUV CL from the Nd^3+^ : LuF_3_ thin film was emitted through the substrate. A substrate with high transmittance in the VUV region was needed to output light efficiently and MgF_2_, which exhibited 94% transmittance at 180 nm, satisfied this condition. The lamp benefited from a low power consumption and reduced thermal effects when the field electron emitters were used as cold cathodes [33, 34]. The lamp was operated in the vacuum chamber at an average pressure of 8 × 10^−5^ Pa.

## 3. Results and Discussion

The thickness and surface morphology of the Nd^3+^ : LuF_3_ thin film was investigated by using scanning electron microscopy (SEM). The thin film contained some droplets with cracks that originate from structural phase transitions. In contrast, the uniform layer was about 15 nm thick without any cracks. The crystallographic properties were also evaluated by using X-ray diffraction. The high and sharp diffraction patterns indicated the well crystallization of the thin film. The detailed data of these evaluations are described in [29].

**Figure 2:**
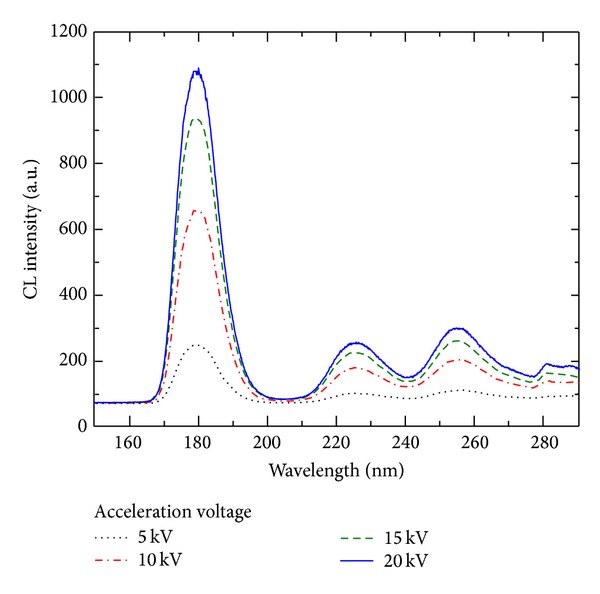
CL spectra of the Nd^3+^ : LuF_3_ thin film at acceleration voltages ranging from 1 to 20 kV.

Figure 2 shows the CL spectra of the Nd^3+^ : LuF_3_ thin film at different acceleration voltages ranging from 1 to 20 kV. The electron beam current was kept at 600 pA during the CL measurements. The spectra showed a dominant peak in the VUV region at 179 nm and two additional emission peaks at 223 and 255 nm, which are consistent with the emission peaks observed for Nd^3+^ : LuF_3_ single crystals [17]. These results show that although the PLD target was obtained by pressing NdF_3_ and LuF_3_ powders together (undoped material), Nd^3+^ acted as a dopant for LuF_3_ and a luminescent center in the thin film.

**Figure 3:**
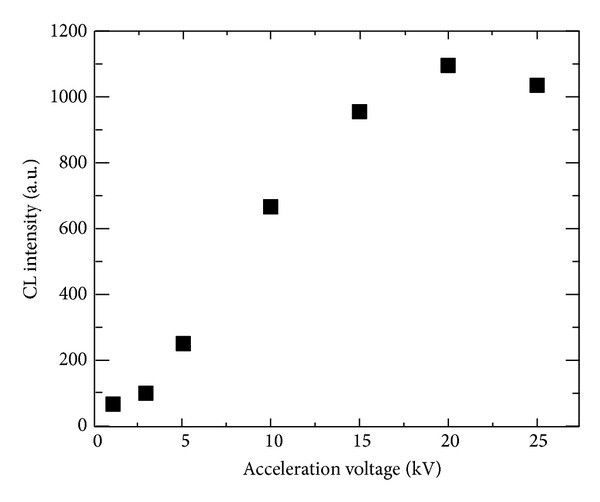
Output CL intensity of the Nd^3+^ : LuF_3_ thin film at 179 nm for acceleration voltages ranging from 1 to 25 kV.

The influence of the acceleration voltage on the CL intensity of the Nd^3+^ : LuF_3_ thin film at 180 nm was also investigated as shown in Figure 3. The CL intensity increased with increasing acceleration voltage before saturation at 25 kV. This result suggests that incident electrons passed through the thin film before giving all their energy to the thin film at 25 kV.

**Figure 4:**
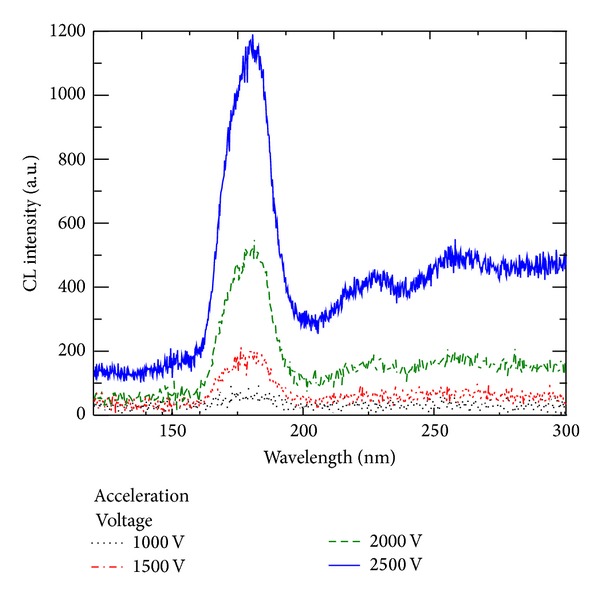
Emission spectra of the lamp at acceleration voltages ranging from 1 to 2.5 kV.

The emission spectra of the lamp were measured at different acceleration voltages ranging from 1 to 2.5 kV. The extraction voltage was kept at 600 V during the measurements. The emission spectra (Figure 4) presented a dominant peak in the VUV region at 180 nm and two additional peaks at 225 and 255 nm. These spectra closely matched the emission spectra obtained for the Nd^3+^ : LuF_3_ thin film.

**Figure 5:**
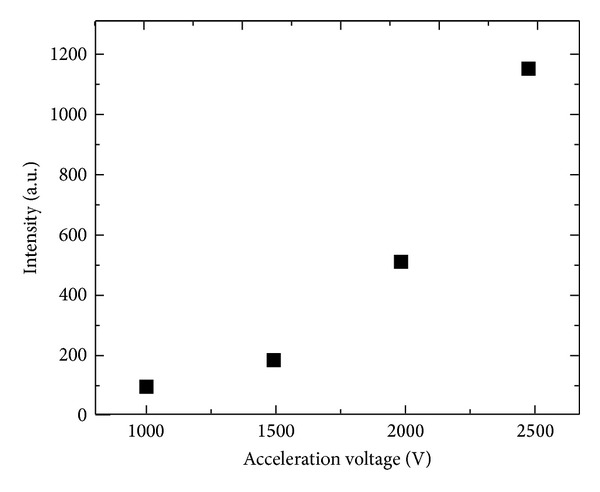
Output CL intensity of the lamp at 180 nm for acceleration voltages ranging from 1 to 2.5 kV.

The influence of the acceleration voltage on the CL intensity of the lamp at 180 nm was evaluated. The CL intensity (Figure 5) showed a nonlinear dependence on the acceleration voltage, which was attributed to an increase of the electron diffusion region in the thin film. The output power of this lamp may amount to several microwatts because Nd^3+^ : LuF_3_ and KMgF_3_ show quasiequivalent conversion efficiencies [16]. An increase in acceleration voltage may therefore efficiently enhance the output power of this lamp.

The luminescence area of this VUV lamp can easily generate a large area with little thermal effect and low power consumption by employing a CNF field electron emitter. In addition a solid-state phosphor brings many benefits in the VUV lamp such as safety, longevity, stability, and downsizing.

## 4. Conclusions

In summary, a VUV field emission lamp consisting of a Nd^3+^ : LuF_3_ thin film as a solid-state phosphor and CNF field electron emitter was fabricated. The CL spectra of the lamp showed multiple emission peaks at 180, 225, and 255 nm, which were in good agreement with emission spectra previously reported for the Nd^3+^ : LuF_3_ crystal. This result suggested that Nd^3+^ ion acted as a luminescent center and doped LuF_3_ in the synthesized thin film although the target used during PLD was obtained by pressing NdF_3_ and LuF_3_ powders into a pellet. Furthermore, the output emission intensity showed a nonlinear response to the acceleration voltage, indicating that an increase in acceleration voltage may significantly enhance this output emission intensity. Although recent gas lamps are improving their performances, this lamp may soon become one of the candidates of VUV light sources. These techniques are essential to numerous applications, such as sterilization, surface cleaning, and synthesis and degradation of chemical material.
